# Primary Health Care and Tuberculosis Detection during the COVID-19 Pandemic: Crucial Actions for Intensifying Efforts

**DOI:** 10.3390/ijerph21050540

**Published:** 2024-04-25

**Authors:** Stephanie Ribeiro, Erika Mayumi Takahashi, Katia Lacerda de Souza, Thais Tiemi Yamamoto, Raquel Russo Leite, Hugo Fernandes, Meiry Fernanda Pinto Okuno, Maria Rita Bertolozzi, Tânia Maria Ribeiro Monteiro de Figueiredo, Roxana Isabel Cardozo Gonzales, Paula Hino

**Affiliations:** 1 Department of Collective Health, Paulista School of Nursing, Federal University of São Paulo, São Paulo 04024-002, São Paulo, Brazil; erika.takahashi@unifesp.br (E.M.T.); hugo.fernandes@unifesp.br (H.F.); mf.pinto@unifesp.br (M.F.P.O.); paula.hino@unifesp.br (P.H.); 2 Municipal Health Secretariat of São Paulo, Health Surveillance Coordination, São Paulo 01223-010, São Paulo, Brazilrachelrusso@prefeitura.sp.gov.br (R.R.L.); 3 Department of Public Health Nursing, School of Nursing, University of São Paulo, São Paulo 05403-000, São Paulo, Brazil; mrbertol@usp.br; 4 Department of Nursing, State University of Paraíba, Campina Grande 58109-753, Paraíba, Brazil; taniaribeiro@servidor.uepb.edu.br; 5 Faculty of Nursing, Federal University of Goiás, Goiânia 74605-080, Goiás, Brazil; roxanaisabel@ufg.br

**Keywords:** public health, tuberculosis, coronavirus, primary health care, health vulnerability, health services research

## Abstract

Background: Tuberculosis has been considered a global emergency since 1993, and controlling it has become even more challenging since 2020 due to the health and social crisis resulting from the COVID-19 pandemic. This study aimed to identify the impact of the COVID-19 pandemic on tuberculosis case detection activities within primary health Care in the largest city in Brazil. Methods: This is a cross-sectional and analytical study on the provision of tuberculosis detection actions in primary healthcare units during the pandemic period. A descriptive analysis was performed for characterization, and Generalized Mixed Models were used for evaluating associations, with a Bonferroni post hoc test applied. Results: The study sample comprised 80 health units in the municipality. There was a moderate alteration level in the provision of consultations for individuals with signs and symptoms of tuberculosis in 2020 (13.8%) and in 2021 (15.1%). Statistical significance (*p* < 0.05) was found between the pandemic period and detection actions, with a lower alteration level in 2022. Conclusions: Tuberculosis detection activities underwent changes due to the COVID-19 pandemic, mainly in 2020, which was associated with alterations in consultation and case notification provision.

## 1. Introduction

Tuberculosis (TB) is an age-old infectious disease with a chronic course and is caused by the *Mycobacterium tuberculosis* complex. Despite being curable in almost all cases treated appropriately and with a considerable reduction in transmission shortly after treatment initiation, it still has one of the highest global mortality rates [1,2].

TB has been considered a global emergency since 1993, and proposals to combat this historical disease have been established internationally. The goal of eliminating TB by 2030 was established through the Sustainable Development Goals (SDGs) set by the United Nations (UN); this reflects the international recognition of the humanitarian crisis surrounding TB, as it predominantly affects vulnerable social groups in underdeveloped countries, with significant economic impact [1,3].

According to the World Health Organization (WHO), it is estimated that 10.6 million people worldwide fell ill with TB in 2022. The estimated incidence in the two previous years was 5.8 million cases (2020) and 6.4 million cases (2021). This means that the year 2022 saw an increase in cases by 82% and 65% when compared to 2020 and 2021, respectively. Persisting as a global public health problem, TB accounted for approximately 1.3 million deaths in 2022 [2].

With an incidence of 87% in low- and middle-income countries, TB also poses a challenge to public health in Brazil, being included in the WHO’s global lists of countries with the highest disease burdens and TB–HIV coinfection [2,4]. The incidence rate of the disease for the year 2022 was 36.3 cases per 100,000 inhabitants, with a mortality rate of 2.3 cases per 100,000 inhabitants in 2021. The state of São Paulo accounted for 22% (17,865) of the country’s cases, with the state capital accounting for 36% of these notifications [4].

Furthermore, the control of TB became even more challenging from 2020 onwards due to the health and social crisis resulting from the COVID-19 pandemic. Isolation and/or social distancing measures were implemented to contain the spread of the disease, and health services were required to reorganize to meet the growing needs of the population, which had impacts on previously developed actions, including those aimed at TB [5,6,7].

A literature review aimed at identifying the impacts on TB control demonstrated a reduction in human resources and restructuring of services during this period. Furthermore, this study found that there was a significant allocation of investments in hospital and private care in Brazil, neglecting the potential of primary health Care (PHC) as a strategic system of the Unified Health System [5]. There was a decrease in TB detection and prevention activities from the centralization of health actions in COVID-19 control, negatively impacting TB control efforts due to the COVID-19 pandemic and deepening social vulnerability. It is important to emphasize that the reduction in TB control activities may reflect a future increase in drug-resistant TB [5,8,9].

Regarding the impact of the COVID-19 pandemic on TB-related actions, a decrease of 18% in TB diagnoses between 2019 and 2020 [10] was identified as a result of the COVID-19 pandemic. Identification of new TB cases in Brazil followed global trends, with a significant decrease in diagnosis during the same period indicated above, approximately 13% [4].

Thus, understanding the growing social inequality with repercussions on TB vulnerability considering the epidemiological indicators of the disease, along with the inevitable restructuring of PHC to address COVID-19, justifies the importance of this study. It aims to identify the impact of the COVID-19 pandemic on TB case detection actions within PHC in the municipality of São Paulo, Brazil.

## 2. Materials and Methods

### 2.1. Study Design

This is a cross-sectional study focusing on TB detection actions during the pandemic period (2020–2022) conducted in primary care services in the municipality of São Paulo. This study was developed in accordance with the Strengthening the Reporting of Observational Studies in Epidemiology (STROBE) guidelines [11].

### 2.2. Study Setting

The municipality of São Paulo had a population of over 11 million inhabitants and a demographic density of 7528.26 individuals/km^2^ [12] in 2022, with Primary Care coverage at 71.9% and Family Health Strategy coverage at 47.4%. The health services included in this study were the 468 health units registered on the official website of the municipal government [13]. The municipality is divided into six Regional Health Coordinators: Central, East, North, West, Southeast, and South. Health services are distributed within these regions, forming part of the TB care network coordinated by the Health Surveillance Coordinator, which includes the Municipal TB Control Program [14].

### 2.3. Study Population and Sampling Design

The study population consisted of healthcare professionals directly involved in TB control actions, referred to as key informants, who were nominated by the manager or the health unit team to participate in this study. Inclusion criteria included being active in PHC services and directly involved in TB control. Health units that had no TB cases during the pandemic period were excluded.

All health units integrated into the PHC comprised the study universe (*N* = 468). A sample calculation was performed to determine the sample size, considering a 10% absolute error value for the estimate implemented by the following formula:

$$n=\frac{{z^{2}}_{\alpha /2}Np\left(1-p\right)}{d^{2}\left(N-1\right)+{z^{2}}_{\alpha /2}p\left(1-p\right)}$$

in which *N* represents the total number of health units in the municipality, *p* represents the estimated proportion of the outcome, and *d* represents the absolute error (or precision) of the estimate. The proportion value (*p*) was considered to be 50%, as this value maximizes the sample size, thereby resulting in a total of 80 health units.

These were selected through stratified random sampling with proportional allocation, where strata corresponded to the municipality’s geographical regions. Health units were then numbered by the health coordinator, and the sample units were randomly selected using the RStudio software program version 4.1.3 (Boston, MA, USA) with the function sample (replace = FALSE).

### 2.4. Variables

The study variables were selected from the tool used in the multicentric project named “Impact of the COVID-19 Pandemic on TB in Brazilian Capitals: Reality and New Perspectives in Primary Care”, to which this study is linked.

The tool was developed on the Research Electronic Data Capture (REDCap) platform version v9.1.0 (Nashville, TN, USA), integrated into the Internet, and used by interviewers during face-to-face data collection. Information was recorded using the REDCap mobile tool.

The data collection tool comprises a structured form, containing questions with multiple-choice, dichotomous, and Likert scale response patterns consisting of three blocks of variables: I. Characteristics of PHC units; II. Characteristics of PHC units focused on TB care; III. TB-related health actions (case detection actions).

The variables selected for this study were those from the block of characteristics of PHC units (independent variables) and the block of TB-related health actions (dependent variables).

### 2.5. Data Collection

Data collection took place from December 2022 to March 2023. The research team conducting data collection received specific prior training to conduct interviews and use the REDCap application.

Health unit managers were contacted to inform them about the research and its ethical and methodological aspects, seeking consent to conduct the study. At this point, they were also asked to nominate a professional to participate in the study as a representative of the PHC unit and to schedule a face-to-face interview according to the participant’s availability. The interview took place in person at the health unit’s premises after presenting and obtaining agreement with an informed consent form.

### 2.6. Data Analysis

The primary source database generated by the REDCap tool was imported as a spreadsheet into Excel^®^ for storing the study data.

The TB case detection action variables presented with Likert scale responses were categorized into four alteration categories: no change (0), low (1–3), moderate (4–7), and high (8–10). This categorization was based on studies evaluating the performance of PHC in TB control [15].

The characterization of PHC units and TB control actions by primary source was conducted through descriptive statistical analysis (absolute and relative frequencies).

Considering the characteristics of PHC units and TB detection actions, hypotheses of association were evaluated using Generalized Mixed Models (GMMs) with Gaussian distribution and a linear link function, which were chosen based on the best fit to the model’s quality parameters.

Based on the significance presented in the GMMs, post hoc Bonferroni tests were used to compare the mean alterations of detection actions for years (2020, 2021, and 2022), regional health coordinators (center, north, south, east, west, and southeast), type of unit (family health unit, traditional basic unit, mixed unit, and others), changes in work processes (0–10), increased working hours (yes and no), and provision of telemonitoring for TB care (yes and no).

A statistical significance level of less than 5% (*p* < 0.05) was adopted. The Jamovi version 2.3.28 (Sydney, NSW, Australia) and RStudio version 4.1.3 statistical programs were used for data analysis.

Independent variables in this study included unit characterization variables classified into Likert scale responses (0–10), discrete (2020, 2021, and 2022), and dichotomous categorical (yes and no), as well as multiple-choice. Outcome variables (TB detection actions) were classified into Likert scale responses (0–10).

### 2.7. Ethical Considerations

This study adhered to the guidelines outlined in Resolution No. 466/2012 of the National Health Council, obtaining approval from the Research Ethics Committee of the sponsoring institution (opinion No. 5,671,976) and the Municipal Health Department of São Paulo (opinion No. 5,767,166).

## 3. Results

The study sample consisted of 80 units from the six Regional Health Coordinators in the municipality of São Paulo, with 1 from the Central (2%), 16 from the North (20%), 22 from the South (27%), 20 from the East (25%), 5 from the West (6%), and 16 from the Southeast (20%). The characteristics of the establishments highlight the prevalence of family health units (53.8%), followed by traditional basic units (32.5%) (Table 1).

In the years 2020 and 2021, the majority reported a high alteration level in work processes (88.9% and 71.3%) and an increase in professionals’ working hours (78.8% and 77.5%), respectively. It was observed that more than 90% of the units underwent changes in the organization of physical space in all the years evaluated, and more than 50% offered telemonitoring for people with TB.

Furthermore, 15% reduced the offer frequency of consultations for people with signs and symptoms of tuberculosis, 8.8% offered remote consultation care for people with signs and symptoms of tuberculosis, and 5% presented an increase in the notification time for tuberculosis cases.

The changes scale in TB detection actions are presented in Table 2. It was observed that 13.8% and 15.1% of health units showed a moderate alteration level in offering consultations for individuals with signs and symptoms of TB in 2020 and 2021, respectively.

Regarding changes in the request for exams, specifically for sputum examinations, a moderate change level was observed in 2020 (13.3%), followed by low changes in 2021 (8.8%) and 2022 (6.3%). There was a moderate change level in relation to chest X-rays in 2020 (12.5%) and 2021 (11.3%) and a low change level in 2022 (2.5%). Moreover, there was a moderate change level for the notification of TB cases during the COVID-19 pandemic in 2020 (3.8%) and in 2021 (3.8%), and a low level in 2022 (2.5%).

When assessing the effects of the COVID-19 pandemic on TB detection actions (0–10) in health units, presented in Table 3, it was observed that the year showed a significant association with the change in providing consultations for respiratory symptomatic individuals (F_2;172.2_ = 4.64; *p* = 0.011) and changes in the notification of TB cases (F_2;169.6_ = 4.15; *p* = 0.017); increased working hours were associated with the change in requesting chest X-rays for diagnosis (F_1;212.8_ = 3.984; *p* = 0.047).

Therefore, the pandemic period had repercussions on providing consultations for respiratory symptomatic individuals and the notification of TB cases. It is noteworthy that the year 2022 showed a lower change level compared to 2020 and 2021 for the two mentioned actions (Table 4). Meanwhile, units reporting an increase in working hours exhibited lower change levels in the request for chest X-rays for diagnosis compared to units that did not report an increase in working hours.

## 4. Discussion

With the aim of identifying the repercussions of the COVID-19 pandemic on TB detection actions in the PHC services of the municipality of São Paulo, this study highlighted changes in both organizational aspects and human resources, noting a decrease in the availability of consultations for respiratory symptoms of TB.

The imposed reorganization of health services and social distancing measures adopted to mitigate the exponential increase in individuals infected with the novel coronavirus generated direct impacts on PHC services. These changes included discontinued care and the interrupted provision of routine care to the population [16].

In the first year of the pandemic, an ecological study aiming to explore its impact on non-COVID-19-related health procedures demonstrated that actions developed within the Brazilian Unified Health System, such as medical consultations, screening, and diagnostic tests, experienced a one-quarter decline, with a significant impact on federative units exhibiting greater socioeconomic vulnerability [17].

Furthermore, similar changes occurred in the routine of PHC services worldwide, as declines in primary care consultations have been observed in countries such as South Africa, Bosnia and Herzegovina, Sweden, and China, among others [18,19,20,21,22].

Such reductions reflect changes in the previously consolidated work processes, which were also identified in this study throughout the pandemic period (2020–2022). The main modification adopted by the city government in the municipality of São Paulo concerned the service flows in health units, based on governmental guidelines aimed at strengthening the units in actions against COVID-19 [23].

A partial and temporary suspension of routine consultations, exams, and procedures was established in March 2020 [24], emphasizing the changes found in this and other Brazilian municipalities [25,26,27]. In this sense, the Ministry of Health in Brazil, reinforced the resolution role of these services in addressing the pandemic through the Clinical Management Protocol for Coronavirus (COVID-19) in PHC [28].

A measure regarding changes in the organization of physical space within the units was adopted to enable new care flows, aiming to minimize contact between users with and without symptoms suggestive of COVID-19 [23]. As a result, PHC services provided access to and special rooms for these consultations [29]. Such measures were equally adopted in Brazil [23,25,26] and corroborate the findings of this study.

Furthermore, it is noted that the increase in working hours resulted in an increased demand for chest X-rays, with delays in radiological and imaging exams reported by several countries [30,31].

Thus, it is evident that PHC underwent concerning changes in its organization during the pandemic period, especially in its first year, with a reduction in important health prevention and promotion programs. These transformations intensified aspects related to vulnerability, manifested in the difficulty of accessing services and health actions, as well as in relation to the quality of service provided, thus weakening the principles of the Brazilian health system.

Actions to control TB also underwent significant changes with the reorganization of health services. The results indicate a decrease in the availability of consultations for respiratory symptoms of TB (15%). Such a change impacts early diagnosis, leading to increased disease burden and social inequalities [5,16].

The global trend of decreased TB case notifications in 2020 (5.8 million) and 2021 (6.4 million), with a recovery in 2022 (7.5 million), contrasts with the year 2019 (7.1 million) [2]. Epidemiological data in Brazil indicate an 8.3% reduction in notifications of new TB cases in 2020, and this reduction was even greater for the state of São Paulo, reaching 11.2% [32].

It was also observed that regions with high notification rates of COVID-19 exhibited lower TB notification rates [33,34], which certainly had an impact on the municipality of São Paulo, accumulating over 2.4 million confirmed cases of COVID-19 between 2020 and 2022 [35]. Another important aspect that likely contributed to the construction of this scenario was the fear present in the population to attend PHC services due to apprehension about contracting the novel coronavirus infection [36,37,38].

These findings undoubtedly demonstrate that TB-related actions faced the consequences of the pandemic period, particularly in the years 2020 and 2021, where a significant association was observed with changes in the availability of consultations for respiratory symptoms and alterations in TB case notifications. These findings corroborate data in the literature, as there were significant reductions in the investigation and notification of TB cases [34,39].

In this health crisis scenario, impacts of reduced TB care were reported in African and Asian continents [20,34,38,40]. A general decrease of 21% in TB diagnoses was observed in antiretroviral therapy clinics serving people living with HIV/AIDS on both continents in the first year of the pandemic compared to the previous year [39]. These findings corroborate the results indicating that the year 2020 showed the greatest changes for TB care, with a 27.5% decrease in consultations for individuals with TB symptoms and a 7.5% decrease in TB case notifications.

A nationwide ecological study in Brazil aimed at exploring the impacts of the pandemic on TB diagnosis in 2020 identified a progressive reduction in cases identified starting from March. There was a significant reduction in positive bacilloscopies (17.1%), particularly in the Southeast (17.6%), Northeast (19.6%), and Midwest (26.5%) regions [32].

These changes represent a setback in the progress achieved and aimed for in relation to Sustainable Development Goal 3.3 and the End TB Strategy, making the path to achieving it even more challenging and costly [2,32]. The decline in TB testing and diagnosis perpetuates a scenario of significant vulnerability, intensifying the population’s susceptibility to TB infection and illness, maintaining its transmission chain.

This situation poses a significant challenge for developing and underdeveloped countries, primarily due to persistent economic, social, and health inequalities. Emergencies and crises such as the COVID-19 pandemic tend to accentuate existing inequalities in society, leading to an increase in unemployment, impoverishment, and food insecurity [41].

It is therefore necessary to implement strategies to mitigate the impacts caused by critical contexts, such as a pandemic, in order to prevent the interruption of TB care services; these strategies can vary from health education actions to laboratory diagnostic and imaging services, providing inputs, training, and support to professionals, and aiming to maintain care provision for both symptomatic respiratory individuals and their contacts [42].

Although changes in TB detection actions were observed due to the COVID-19 pandemic, the majority were of a low level, showing a difference of less than 10% on the Likert scale, concerning the characteristics of health units and the years related to TB detection actions.

There is one limitation that can be highlighted in understanding the relevance of the results in this current study regarding the impact of the COVID-19 pandemic on TB case detection activities: the admission of a 10% estimation error to determine the sample size and only interviewing one healthcare professional from each APS unit. Nonetheless, the importance of the information provided by the participants is emphasized, as they were designated as key informants for this study, and the sample size does not undermine the findings of this study, which have the potential to positively contribute to controlling TB.

The presented data can significantly contribute to improving TB care provision, as they provide a situational diagnosis of the TB scenario within the context of the COVID-19 pandemic. In turn, they offer support for strengthening the municipal TB control program, thus contributing to the elimination of the disease.

## 5. Conclusions

This study presents evidence that TB detection actions in the city of São Paulo underwent changes during the COVID-19 pandemic, as did PHC services. From the results, it was evident that there was a reduction in the number of consultations for individuals with signs and symptoms of TB, especially in 2020.

The importance of diagnosis for controlling the disease is emphasized, and government efforts will be necessary to expand detection actions aiming to reverse the impacts of the pandemic and achieve elimination of TB as a public health problem.

## Figures and Tables

**Table 1:** Characteristics of health units (*n* = 80), São Paulo-SP, 2020–2022.

Variable		*n*	%
Type of unit			
Family Health Unit		43	53.8
Traditional Basic Unit		26	32.5
Mixed Unit		10	12.5
Other		1	1.3
Change in the organization of the physical space of the unit			
2020	Yes	78	97.5
2021	Yes	77	96.3
2022	Yes	72	90
Change in the work process			
2020	No change	0	0
	Low	1	1.2
	Moderate	8	10.1
	High	71	88.9
2021	No change	0	0
	Low	1	1.2
	Moderate	22	27.6
	High	57	71.3
2022	No change	6	7.5
	Low	9	11.4
	Moderate	39	48.8
	High	26	32.6
Increase in professionals’ working hours			
2020	Yes	63	78.8
2021	Yes	62	77.5
2022	Yes	39	48.8
Offer of telemonitoring for individuals with tuberculosis			
	Yes	44	55
Reduction in the frequency of consultations for people with signs and symptoms of tuberculosis			
	Yes	12	15
Offering remote consultation care for people with signs and symptoms of tuberculosis			
	Yes	7	8.8
Scheduling appointments for people with signs and symptoms of tuberculosis			
	Yes	2	2.5
Increased notification of new tuberculosis cases			
	Yes	1	1.3
Failure to report tuberculosis cases			
	Yes	2	2.5
Increase in the notification time for tuberculosis cases			
	Yes	4	5

**Table 2:** Changes in tuberculosis detection actions carried out in primary healthcare for individuals with tuberculosis symptoms (*n* = 80), São Paulo-SP, 2020–2022.

Variable		No Change		Low Level		Moderate Level		High Level	
		(0)		(1–3)		(4–7)		(8–10)	
		*n*	%	*n*	%	*n*	%	*n*	%
Change in the provision of consultations for individuals with signs and symptoms of TB	2020	58	72.5	3	3.8	11	13.8	8	10
	2021	58	72.5	5	6.3	12	15.1	5	6.3
	2022	70	87.5	5	6.3	4	5	1	1.3
Change in the request for sputum examinations for diagnosis	2020	63	78.8	1	1.3	11	13.8	5	6.3
	2021	63	78.8	7	8.8	6	7.5	4	5
	2022	71	88.8	5	6.3	3	3.8	1	1.3
Change in the request for chest X-rays for diagnosis	2020	66	82.5	1	1.3	10	12.5	3	3.8
	2021	66	82.5	3	3.8	9	11.3	2	2.5
	2022	75	93.8	2	2.5	2	2.5	1	1.3
Change in the notification of cases	2020	74	92.5	1	1.3	3	3.8	2	2.5
	2021	76	95	0	0	3	3.8	1	1.3
	2022	77	96.3	2	2.5	0	0	1	1.3

**Table 3:** Association between the pandemic period and unit characteristics regarding tuberculosis detection actions, São Paulo-SP, 2020–2022.

Variable	Years		Regional Health Coordinators		Type of Unit		Changes in Work Processes		Increased Working Hours		Provision of Telemonitoring for TB Care	
	F (df)	*p*	F (df)	*p*	F (df)	*p*	F (df)	*p*	F (df)	*p*	F (df)	*p*
Change in the provision of consultations for individuals with signs and symptoms of TB	4.6453 (2; 172.2)	0.011 *	0.5675 (5; 70.7)	0.725	0.3604 (2; 71.7)	0.699	1.165 (1; 219.2)	0.282	0.0481 (1; 208.7)	0.827	0.04729 (1; 69.28)	0.828
Change in the request for sputum examinations for diagnosis	2.068 (2; 175.3)	0.13	1.4421 (5; 70)	0.22	0.1196 (2; 71.1)	0.887	2.0712 (1; 224.7)	0.151	0.0171 (1; 218.2)	0.896	0.22211 (1; 68.48)	0.639
Change in the request for chest X-rays for diagnosis	2.808 (2; 173.7)	0.063	1.262 (5; 70.7)	0.29	0.165 (2; 71.8)	0.848	3.27 (1; 222.1)	0.072	3.984 (1; 212.8)	0.047 *	0.1136 (1; 69.27)	0.737
Change in the notification of cases	4.15 (2; 169.6)	0.017 *	0.692 (5; 71.1)	0.631	0.162 (2; 71.9)	0.851	1.261 (1; 211.4)	0.263	0.62 (1; 200)	0.432	0.06993 (1; 69.67)	0.792

The table includes the associations by GMM. “F” refers to the statistical model for measuring the association between variables. “df” refers to the degrees of freedom, used by the F statistic to measure the association. “*p*” refers to the *p*-value, that is, the proof value of the statistical test. * Indicates that the result is statistical significance (*p* < 0.05).

**Table 4:** Associations between the pandemic period and increased working hours concerning tuberculosis detection actions, São Paulo-SP, 2020–2022.

Variable	Pairwise Comparison	Mean Difference	Standard Error	*p*
Years				
Change in the provision of consultations for individuals with signs and symptoms of TB	2020–2021	0.211	0.243	1
	2020–2022	1.002	0.338	0.01 *
	2021–2022	0.792	0.306	0.032 *
Change in the notification of cases	2020–2021	0.182	0.119	0.388
	2020–2022	0.481	0.168	0.014 *
	2021–2022	0.299	0.152	0.15
Increased working hours				
Change in the request for chest X-rays for diagnosis	No–Yes	0.527	0.266	0.049 *

The table includes the comparison of means by post hoc Bonferroni. “*p*” refers to the *p*-value estimated by the Bonferroni correction, that is, the proof value of the statistical test. * Indicates that the result is statistical significance (*p* < 0.05).

## Data Availability

The datasets presented in this article are not readily available because the data are part of an ongoing study. Requests for access to datasets should be directed to the corresponding author.
